# Synthesis of *O*-Amino Sugars and Nucleosides

**DOI:** 10.3390/molecules23030641

**Published:** 2018-03-12

**Authors:** Na Chen, Juan Xie

**Affiliations:** 1 School of Biomedical Sciences, Huaqiao University, Xiamen 361021, China; nachen@hqu.edu.cn; 2 PPSM, ENS Paris-Saclay, Institut d’Alembert, CNRS, Université Paris-Saclay, 94235 Cachan, France

**Keywords:** sugar aminooxy acid, glycosyl aminooxy acid, *N*-oxyamide, oligosaccharide, glycolipid, glycopeptide, *O*-amino nucleoside, nucleo-aminooxy acid

## Abstract

Nucleic acids and carbohydrates are essential biomolecules involved in numerous biological and pathological processes. Development of multifunctional building blocks based on nucleosides and sugars is in high demand for the generation of novel oligonucleotide mimics and glycoconjugates for biomedical applications. Recently, aminooxyl-functionalized compounds have attracted increasing research interest because of their easy derivatization through oxime ligation or *N*-oxyamide formation reactions. Various biological applications have been reported for *O*-amino carbohydrate- and nucleoside-derived compounds. Here, we report our efforts in the design and synthesis of glyco-, glycosyl, nucleoside- and nucleo-aminooxy acid derivatives from readily available sugars and amino acids, and their use for the generation of *N*-oxyamide-linked oligosaccharides, glycopeptides, glycolipids, oligonucleosides and nucleopeptides as novel glycoconjugates or oligonucleotide mimics. Delicate and key points in the synthesis will be emphasized.

## 1. Introduction

The high nucleophilicity of hydroxylamine derivatives as well as the unique structural and chemical properties of the N-O, oxime and *N*-oxyamide bonds have triggered the *O*-amino functionalization of biomolecules like peptides, proteins, carbohydrates, nucleosides and nucleotides. Due to repulsion between the lone pairs of the electron of the nitrogen and oxygen atoms, the N-O bond of hydroxylamine derivatives has unusual conformational properties, which have been exploited to obtain particular N-O turn structures in aminooxy acid-derived peptides for the preparation of new foldamers, anion receptors or channels [1,2]. The N-O bond is relatively sensitive to radical conditions because of the low N-O bond dissociation energy [3]. Oximes show higher hydrolytic stability than hydrazones [4], while the *N*-oxyamide linkage is resistant to chemical hydrolysis [1]. Aminooxylated carbohydrates and glycoconjugates are attracting the increasing interest for the understanding of the glycobiology and for diverse biomedical applications [3,5]. *N*-, *O*-glycosyl hydroxylamines as well as *N*-hydroxyamino and *O*-amino sugars have been developed over the last twenty years. Various chemically modified oligonucleotides have also been reported, with an increasing number showing up as drug candidates for oligonucleotide therapies [6,7,8,9]. Among them, *O*-amino nucleosides have been prepared for the development of human UDP-GlcNAc 4-epimerase inhibitors [10], modified oligonucleotides [11] or for next generation sequencing [12]. N-O, *N*-oxyamide or *N*-alkoxycarbamate-linked oligonucleotides have also been synthesized as oligonucleotides mimics [13,14,15,16,17]. The focus of this article is to summarize our own efforts in the preparation of *O*-amino sugar and nucleoside derivatives, including sugar aminooxy acids, glycosyl α-aminooxy acids, *O*-aminooxy nucleosides and nucleo-aminooxy acids for the generation of *N*-oxyamide-linked oligosaccharides, glycopeptides, glycolipids, and oligonucleoside mimics.

## 2. Sugar Aminooxy Acids for Oligosaccharide Mimics

The design and synthesis of novel molecules bearing different functions are of great interest for organic, bioorganic, medicinal as well as material chemistry. The creation of cleverly functionalized molecules has a large impact in designing bioactive molecules and for drug discovery. Apart from their biological involvement, carbohydrates are readily available chiral compounds which have been widely used in organic synthesis. We have decided to introduce both aminooxyl and carboxyl functions on the sugar pyranosidic or furanosidic skeletons to generate sugar aminooxy acids or glycoaminooxy acids as multifunctional building blocks which could be used for the synthesis of novel *N*-oxyamide linked oligosaccharides or glycoconjugates with interesting structural properties. It has been shown that *N*-oxyamide-linked peptides can easily form intramolecular hydrogen bonds to facilitate turns and helical structures [1,2]. A rigid ribbon-like secondary structure has been observed on a trimer of a *cis*-β-furanosidic sugar aminooxy acid by the group of Chandrasekhar [18]. Nanorod formation through intermolecular H-bonding has also been observed on a symmetric cyclotetrapeptide prepared from a 2-(*C*-furanosyl) β-amino acid and an α-aminooxy acid [19]. Besides, the *N*-oxyamide-linkage is resistant to chemical and enzymatic hydrolysis [20], and *N*-oxyamide bonds could be readily formed using classical amide formation methods.

**Scheme 1:**
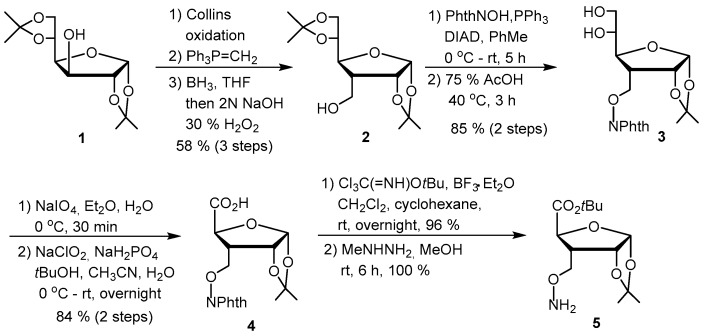
Synthesis of glycofuranosidic aminooxy acids.

We have synthesized d-ribofuranosidic glycoaminooxy acid **4** from diisopropylidene d-glucose (**1**, Scheme 1) [21]. Since initial efforts to introduce an aminooxyl group at the 3-position of **1** failed, probably due to the steric hindrance, we converted **1** into its homologue **2** through Collins oxidation, Wittig reaction, and hydroboration-hydroxylation reactions. Unlike the secondary alcohol function in **1**, the primary alcohol function in **2** reacted efficiently under Mitsunobu conditions with *N*-hydroxyphthalimide to give **3** after selective deprotection of the 5,6-*O*-isopropylidene moiety. Oxidative cleavage of the diol, followed by oxidation of the resulting aldehyde, afforded the desired phthalimidooxyl acid **4** which can be readily converted into aminooxy ester **5**.

**Scheme 2:**
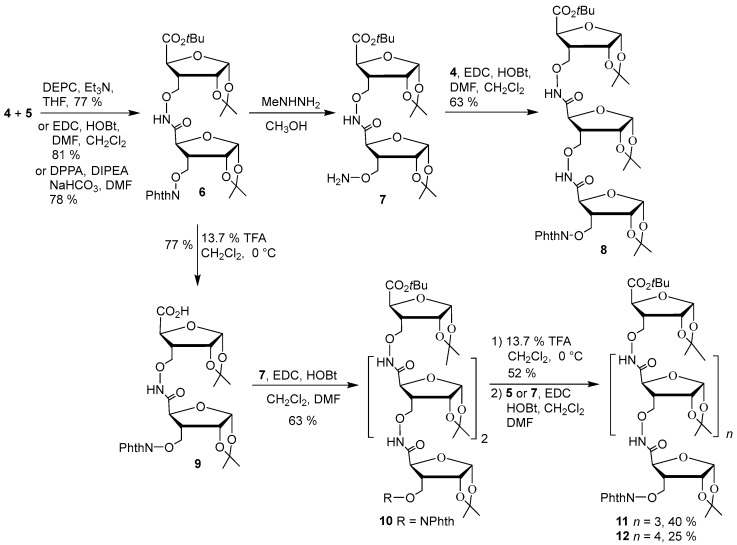
Synthesis of dimers to hexamers of furanosidic glycoaminooxy acid.

The building blocks **4** and **5** were coupled to form oligomers from di- to hexamers (Scheme 2). For the synthesis of oligomers with more than three sugar units, the *t*Bu group was selectively removed using 13.7% trifluoroacetic acid (TFA) in CH_2_Cl_2_ without affecting the acetonide protecting groups. *N*-Oxyamide bond formation can be realized by using *N*-ethyl-*N*’-(3-dimethylaminopropyl)carbodiimide (EDC)/1-hydroxy benzotriazole (HOBt) method or two organophosphorous reagents diphenylphosphoryl azide (DPPA)/*N,N*-diisopropylethylamine (DIPEA)/NaHCO_3_ and diethyl cyanophosphonate (DEPC)/Et_3_N in similar yields.

**Scheme 3:**
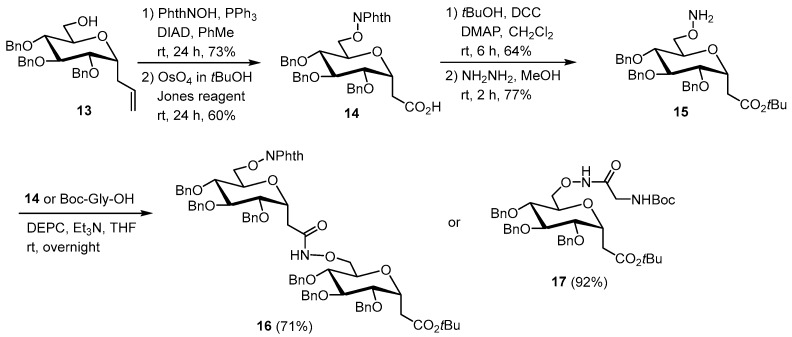
Synthesis of pyranosidic glycoaminooxy acid **14** and conjugates.

Concerning pyranosidic glycoaminooxy acids, we have firstly prepared the compound **14** bearing the carboxylic acid on the *C*-glycosidic anomeric chain (Scheme 3) [22]. This compound could be easily prepared from the readily available *C*-allyl glucoside **13** containing a free hydroxyl group at the C-6 position [23]. Hence, compound **13** was converted to the phthaloyl-protected glycoaminooxy acid **14** in two steps involving Mitsunobu reaction with PhthNOH, followed by oxidation of the alkene moiety to a carboxylic acid. The compound **14** was further transformed into the building block **15** via protection of the carboxylic acid as a *t*Bu-ester followed by the removal of the phthaloyl group to afford the free oxyamine. To demonstrate the synthetic utility of the building blocks **14** and **15** in the preparation of oligosaccharide and glycopeptide mimetics, compound **15** was coupled with **14** or Boc-Gly-OH to give the disaccharide **16** or glycosyl amino acid **17** in good yield.

**Scheme 4:**
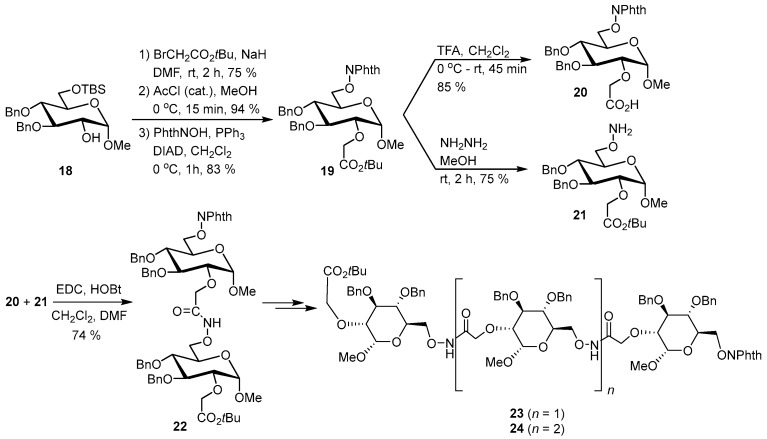
Synthesis of pyranosidic glycoaminooxy acid **20** and oligosaccharide mimics **22**–**24**.

The pyranosidic glycoaminooxy acid building blocks bearing the aminooxyl and carboxylic groups on the 2,6-positions were prepared from the d-glucopyranoside **18** (Scheme 4) [24]. Alkylation of the hydroxyl group with BrCH_2_CO_2_*t*Bu followed by acidic hydrolysis of the *tert*-butyldimethylsilyl (TBS) group and conversion of the resultant hydroxyl to phthalimidooxyl group afforded the fully protected glycoaminooxy acid **19**. Selective removal of the *t*Bu group using TFA in CH_2_Cl_2_ formed the building block **20** bearing a free carboxylic acid. On the other hand, the phthaloyl group was removed through hydrazinolysis to the building block **21**. The two orthogonally protected glycoaminooxy acid units were employed for the preparation of oligomers from dimer **22** to tetramer **24**.

From our results and those reported in the literature [3,5], we can conclude that oligomers derived from sugar aminooxy acids can be obtained as efficiently as those formed from the previously developed sugar amino acid building blocks [25,26,27]. However, benzyl protecting groups are to be avoided due to the sensitivity of the N-O bond towards the benzyl-deprotecting hydrogenolysis conditions [3].

**Scheme 5:**
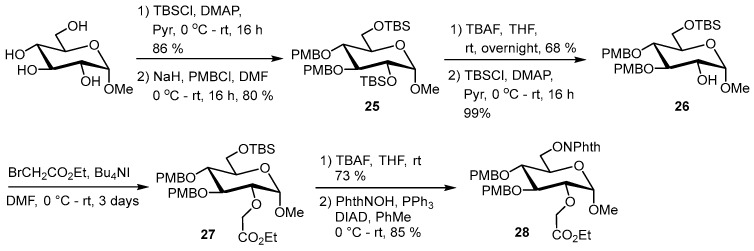
Synthesis of pyranosidic glycoaminooxy ester **28**.

We then decided to use acid-sensitive *para*-methoxybenzyl groups (Scheme 5) [28]. First, methyl α-d-glucopyranoside was selectively protected with TBS and *para*-methoxybenzyl (PMB) groups to **25** and **26**. Contrary to compound **18**, the 2-*O*-alkylation of **26** has necessitated the optimization of the reaction conditions due to the very low conversion. The best conversion (64%) was obtained by slow addition of BrCH_2_CO_2_Et after formation of the alcoolate. The phthalimidooxyl group has been introduced under Mitsunobu conditions after desilylation, leading to the PMB-protected glycoaminooxy ester **28**. The efficient deprotection of the PMB has been demonstrated in the corresponding glycolipids mimics (vide infra).

## 3. Glycosyl α-Aminooxy Acid Derivatives

**Scheme 6:**
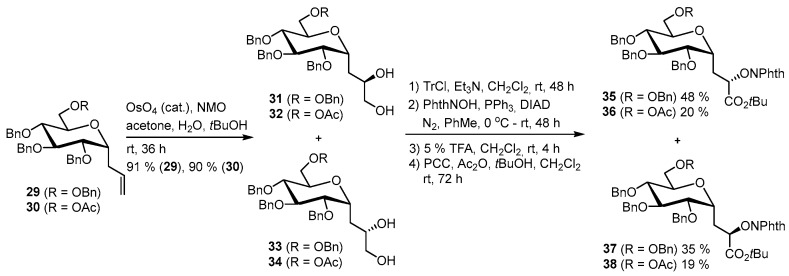
Synthesis of α-*C*-glucosyl α-aminooxy esters.

Glycosyl amino acids are constituents of glycopeptides and glycoproteins playing key roles in biological processes. We have synthesized *C*- and *O*-glycosyl aminooxy acid derivatives as novel glycosyl amino acid building blocks for the generation of glycopeptide mimics. The α-*C*-glycosyl aminooxy acid derivatives can be readily prepared from α-*C*-allyl glucosides **29** and **30**, through dihydroxylation, selective tritylation, Mitsunobu reaction with PhthNOH, detritylation and oxidation [29] (Scheme 6). Both α-*C*-glucosyl l- and d-α-aminooxy esters **35**–**38** have been successfully synthesized.

**Scheme 7:**
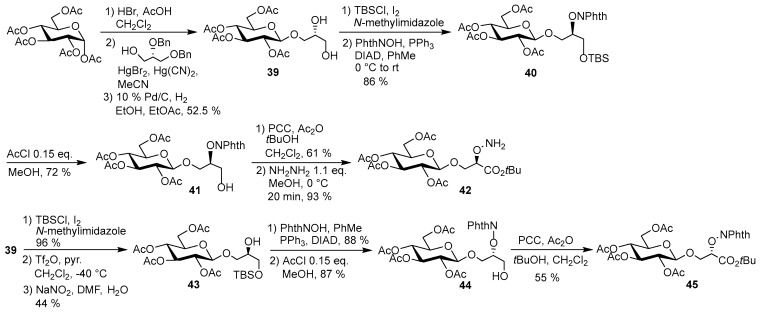
Synthesis of *O*-glycosyl α-aminooxy esters.

For the *O*-glycosyl aminooxy acid derivatives, we have stereoselectively synthesized both (2*R*)- and (2*S*)-aminooxy analogues of β-*O*-glucosylserine from *sn*-(3-β-*O*-glucosyl)glycerol **39** which was obtained through glycosylation of *sn*-1,2-di-*O*-benzyl-glycerol with d-glucose pentaacetate followed by debenzylation (Scheme 7) [30]. Selective silylation and Mitsunobu reaction with PhthNOH and acidic desilylation afforded the *sn*-(2-*O*-phthalimido-1-*O*-glucosyl)glycerol **41** which was transformed into the (2*R*)-3-β-*O*-glycosyl aminooxy ester **42** after selective deprotection of phthaloyl group with 1.1 equivalent of hydrazine in methanol during 20 min at 0 °C. To prepare the (2*S*)-isomer, the configuration of the secondary alcohol was inverted via triflation followed by reaction with NaNO_2_ (Lattrell-Dax epimerization method). The resulting alcohol **43** was converted to (2*S*)-β-*O*-glucopyranosylaminooxyl ester **45** by following the same synthetic sequence employed for the (2*R*)-stereoisomer.

**Scheme 8:**
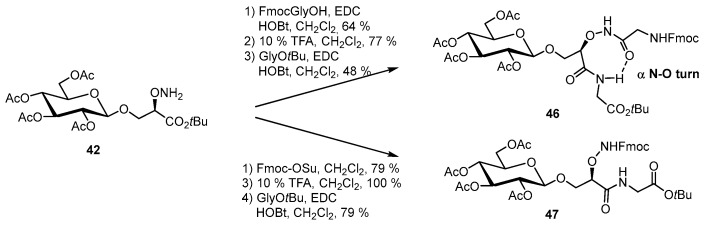
Synthesis of *N*-oxyamide-linked glycopeptides.

With a reliable method to both stereoisomers of the β-*O*-glucosylserine analogues in hand, the synthesis of *N*-oxyamide-linked glycopeptides was investigated (Scheme 8). The glucosyl tripeptide mimetic **46** was prepared from **42** after coupling with FmocGlyOH, deprotection of the *t*Bu group, and coupling with GlyO*t*Bu. The *O*-amino function can also be protected with Fmoc group and the corresponding glycopeptide **47** could be further used in the solid-phase peptide synthesis. Interestingly, NMR and IR studies suggested that compound **46** formed an α N-O turn structure via an eight-membered-ring intramolecular H bond between the amide NH and the carbonyl group of the *N*-oxyamide.

## 4. *N*-Oxyamide-Linked Glycolipids

**Scheme 9:**
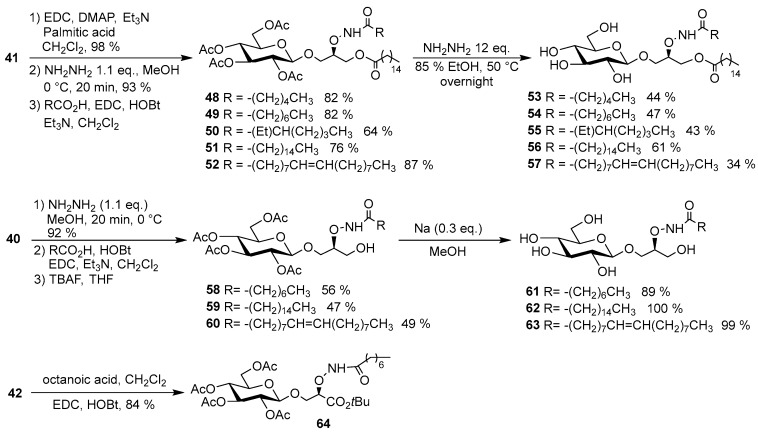
Synthesis of *N*-oxyamide-linked β-glucolipids.

As part of the glycoconjugate family, glycolipids are implicated in a variety of important biological phenomena such as cell-cell interactions, viral and bacterial infections, immune responses, signal transduction, cell proliferation, etc. Natural glycolipids like glycoglycerolipids (GGLs) and glycosphingolipids (GSLs), as well as their synthetic mimics, have also shown very interesting biological activities [31,32]. The design of glycolipid mimics has become a useful strategy in drug discovery. We have prepared *N*-oxyamide-linked glycolipids as mimics of glycoglycerolipids and glycosphingolipids by replacing the ester group in GGL with a *N*-oxyamide functionality [33,34]. The β-glucolipids bearing two lipid chains **53**–**57** were synthesized from the *O*-phthaloylamino β-glucoglycerol **41**, by esterification with palmitic acid, removal of the phthaloyl group and coupling with different carboxylic acids so as to introduce a lipid chain onto the nitrogen atom, and final full deprotection with hydrazine (Scheme 9). Three β-glucolipids bearing one lipid chains **61**–**63** have also been obtained from **40**. Coupling of octanoic acid with the glucosyl aminooxy ester **42** led to glucolipid **64** [30].

**Scheme 10:**
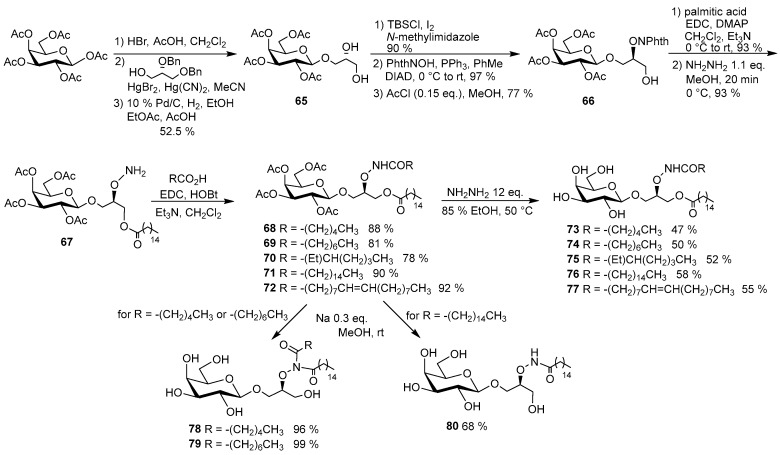
Synthesis of *N*-oxyamide-linked β-galactolipids.

Synthesis of galactoglycerolipids **73**–**77** has been realized from D-galactose pentaacetate in a similar way as the gluco-derivatives (Scheme 10) [34]. Interestingly, deacetylation of **68** and **69** under Zemplén conditions gave the intramolecular transacylation products **78** and **79**, while the compound **71** led to the galactolipid **80**. The synthesized glycolipids bearing one and two palmitic acyl chains **56**, **62**, **76** and **80** are able to assemble with polyethyleneglycol (PEG) thiol-coated gold nanoparticles to form a new type of glyco-Au nanoparticle (AuNP) capable of receptor-targeting cell imaging and drug delivery. The galactolipids derived galacto-AuNPs are selective to galactose-selective peanut agglutinin (PNA), while the gluco-AuNPs selective for concanavalin A (Con A). The efficiency of **76**-AuNP for receptor-targeting hepatocellular imaging of human liver cancer Hep-G2 and the anticancer drug hydrocamptothecin delivery has been demonstrated.

**Scheme 11:**
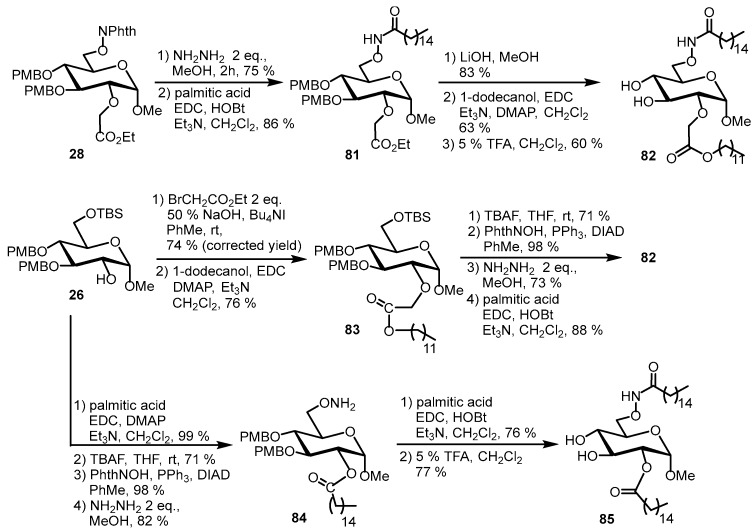
Synthesis of *N*-oxyamide-linked glucolipids **82** and **85**.

Glycolipids bearing lipid chains on the 2,6-positions of methyl α-d-glucopyranoside have been prepared from the 3,4-di-*O*-PMB protected derivatives **26** and **28** (Scheme 11) [28]. Glycolipid **82** is readily obtained from the sugar phthalimidooxy ester **28** after successive deprotection, coupling reactions. The PMB group can be removed by 5% TFA in dichloromethane. Compound **82** is also accessible from **26** after careful 2-*O*-alkylation, followed by usual coupling, deprotection and Mitsunobu reactions. From **26**, we have also prepared glycolipid **85** bearing a 2-*O*-palmitate.

## 5. *O*-Aminooxy Nucleosides

**Scheme 12:**
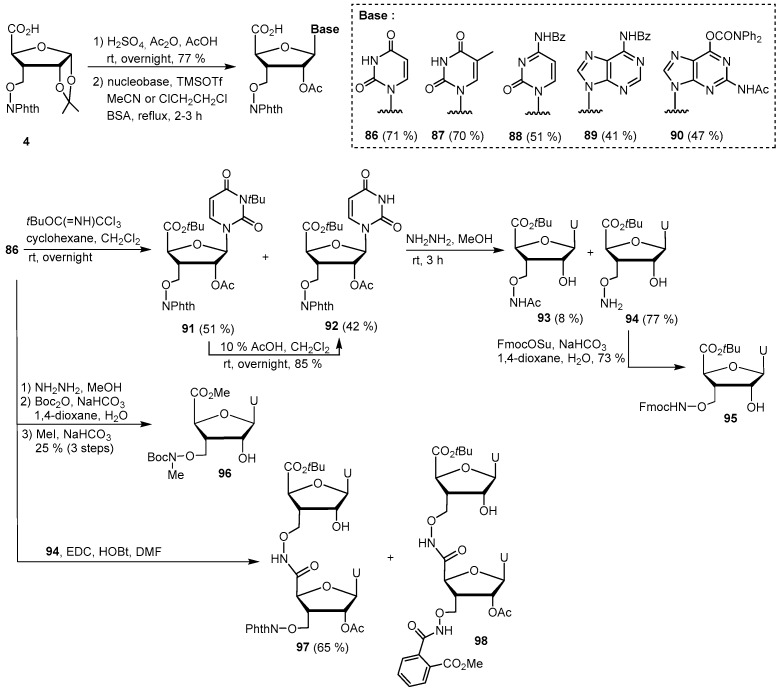
Synthesis of nucleoside aminooxy acids and *N*-oxyamide-linked dinucleoside.

We have designed nucleoside aminooxy acids as novel building blocks for the development of stable oligonucleotide mimetics [35]. Replacement of the ionic phosphodiester by the neutral *N*-oxyamide linkage would not only increase its cellular permeability but also enhance its resistance to extra- and intracellular nucleases degradation. Synthesis of modified RNA oligomers has attracted renewed interest since the discovery of small interfering RNA (siRNA) for gene regulation [6]. We are particularly interested in the development of ribonucleoside aminooxy acid which could be further used for the generation of *N*-oxyamide-linked oligoribonucleosides. From the previously prepared furanosidic glycoaminooxy acid **4**, five nucleoside aminooxy acids **86**–**90** have been synthesized through *N*-glycosylation in the presence of *N*,*O*-*bis*(trimethylsilyl)acetamide (BSA) and trimethylsilyl trifluoromethanesulfonate (TMSOTf) after acidic hydrolysis of the acetonide and acetylation of the resulting hydroxyl groups (Scheme 12). Uridine derivative **86** was chosen for the investigation for *N*-oxyamide-linked oligomer formation. Treatment with the *tert*-butoxytrichloroacetimidate led to the formation of the desired *t*Bu ester **92** along with compound **91** bearing an extra *t*Bu group on the nitrogen atom of the uracil moiety. Compound **91** could be converted to **92** using 10% AcOH in CH_2_Cl_2_. Hydrazinolysis of **92** led to the removal of both phthaloyl and acetyl group to **94**, along with a small amount of transacetylation product **93**. The oxyamine can be further protected with Fmoc group (**95**). Boc protected *N*-methyl nucleoside aminooxy ester **96** can also be prepared from **86**. Coupling of the building blocks **86** and **94** led to the *N*-oxyamide-linked dinucleoside **97**. We have observed the formation of the methanolysis product **98** during the column chromatography with CH_2_Cl_2_/MeOH as eluent. 

**Scheme 13:**
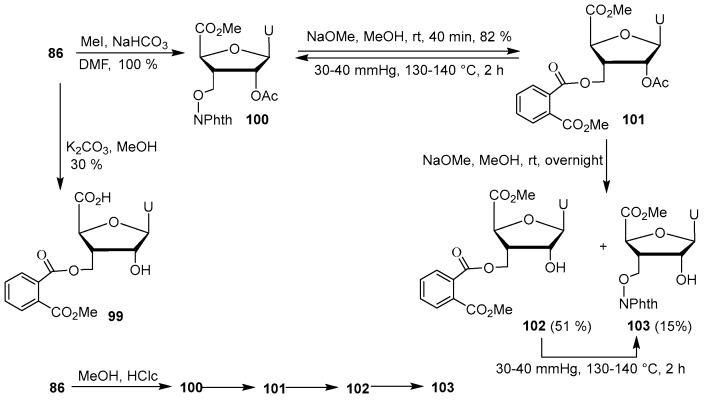
Stability studies of phthaloyl protected nucleoside aminooxy acid.

We then decided to investigate the stability of uridine derivative **86** in the presence of MeOH (Scheme 13). Treatment of **86** with K_2_CO_3_ in MeOH at room temperature led to the ring-opening product **99** in 30% isolated yield. Esterification of **86** in DMF led quantitatively to the methyl ester **100**. Subsequent deacetylation with NaOMe led firstly to the phthalimido ring-opening product **101** which can be converted back to **100** by heating at 130–140 °C under reduced pressure. Further reaction with NaOMe deprotected the 2’-*O*-acetyl group, providing a mixture of compounds **102** and **103**. Similar phenomena have been observed during the saponification of **100** with K_2_CO_3_ or NaOH (4M) in MeOH. The ring closure reaction of the phthalimido group can be achieved by heating **102** under reduced pressure. Under acidic condition in MeOH, we have observed successive transformations of compound **86** to the methyl ester **103** through the intermediates **100** to **102** on thin-layer chromatography (TLC).

**Scheme 14:**
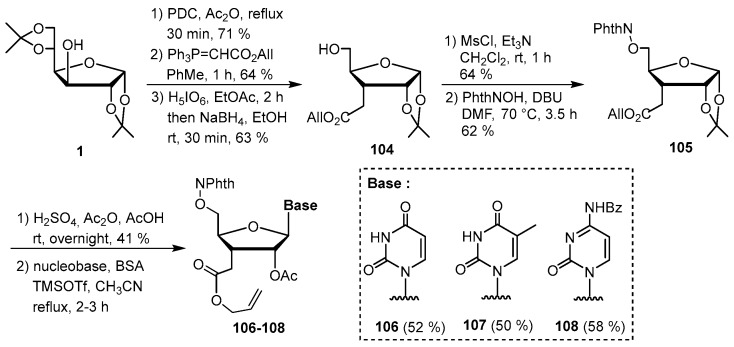
Synthesis of nucleoside aminooxy esters **106**–**108**.

From the diacetone d-glucose **1**, nucleoside aminooxy esters **106**–**108** bearing phthalimidooxyl group on 5’-position have been prepared according to the Scheme 14 [36]. Compound **1** was first converted to allylic ester **104**. Mitsunobu reaction of **104** with PhthNOH led to impure **105** which was then prepared through the mesylate intermediate followed by nucleophilic substitution reaction. *N*-glycosylation in the same conditions as for **4** led to the target nucleosides **106**–**108**.

**Scheme 15:**
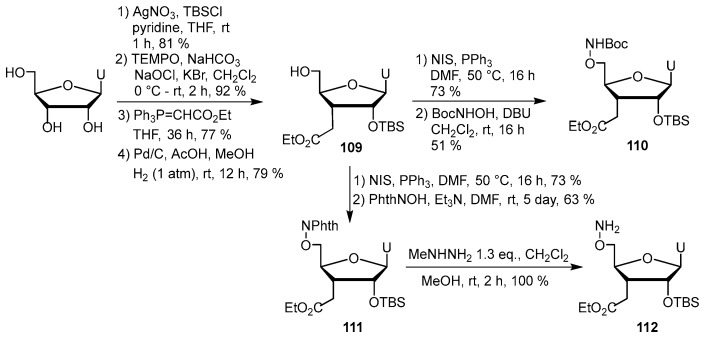
Synthesis of nucleoside aminooxy esters **110**–**112**.

From the uridine, we have prepared the uridine aminooxy esters through selective 2’,5’-di-*O*-silylation, oxidation of 2’-OH followed by Wittig reaction and hydrogenation under acidic condition to give stereoselectively the ester **109** (Scheme 15) [36]. However, all temptations to introduce the phthalimidooxyl function on 5’-position through the Mitsunobu reaction failed. Hopefully, the 5’-hydroxyl function can be converted to Boc- or phthaloyl protected **110** and **111** through iodide intermediate, then deprotected to oxyamine nucleoside **112**. Unfortunately, deprotection of the ester groups in **110** and **111** led to degradation, making further formation of *N*-oxyamide-linked nucleoside aminooxy acid dimers impossible. We have also tried to protect the oxyamine in **112** with a 4-pentenoyl (Pen) group. Once again, decomposition was observed during the saponification or removal of the Pen-protecting group with I_2_ [37].

**Scheme 16:**
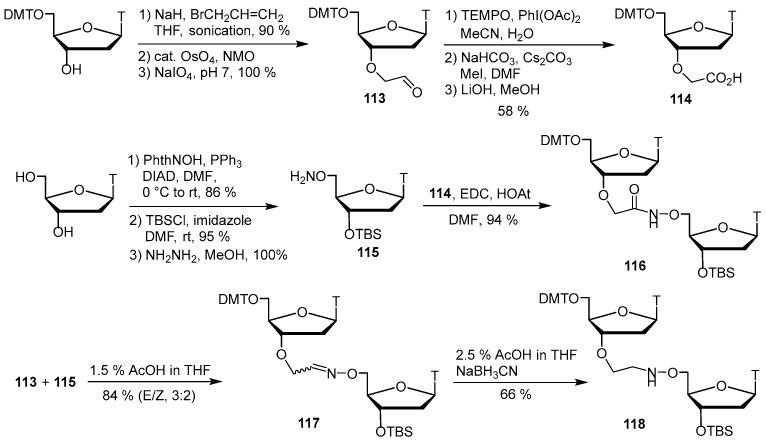
Synthesis of thymidine dinucleosides **116**–**118**.

N-O-linked thymidine dimers were prepared from commercially available thymidine derivatives through introduction of an aminooxyl function on the 5’-position in **115** and an aldehyde or carboxylic functions on 2’ in **113** and **114** (Scheme 16) [38]. Coupling of **114** with **115** led to the *N*-oxyamide-linked dithymidine **116**, while condensation of aldehyde **113** with oxyamine **115** gave the oxime linked dimer **117** as a mixture of *E* and *Z* isomers (3:2) which can be reduced to the oxyamine-linked dinucleoside **118**.

## 6. Nucleo-Aminooxy Acids

**Scheme 17:**
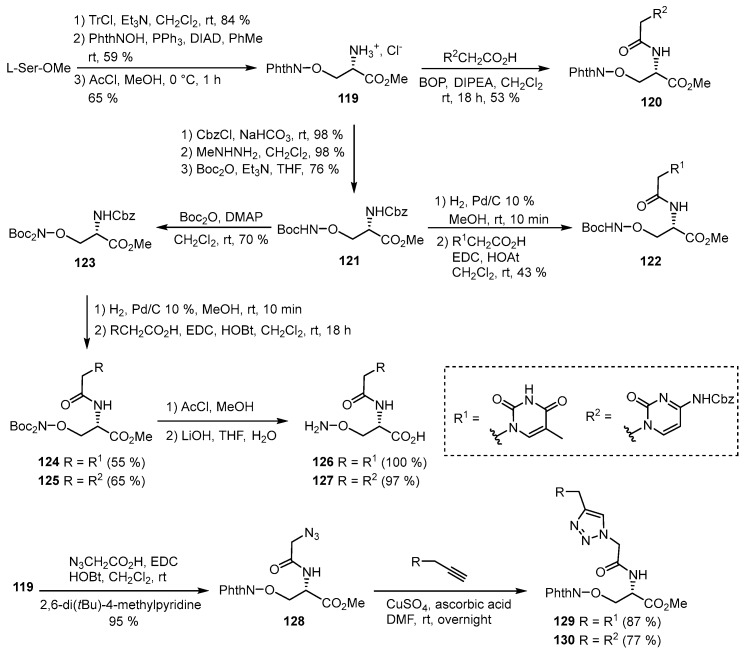
Synthesis of nucleo β-aminooxy acid derivatives.

As promising alternatives to peptide nucleic acids, nucleobase-functionalized peptides have attracted increasing interest because of their well-ordered secondary structures and stability towards enzymatic degradations, as well as their cellular nucleus penetration ability without cytotoxic effects [39,40,41]. We have designed and synthesized α-amino-β-aminooxy acid derivatives bearing nucleobases covalently attached to its α-position through an amide or triazole linkage as novel building blocks of nucleopeptides (Scheme 17) [42]. The key aminooxy acid **119** was prepared from l-Ser-OMe. Coupling reaction with carboxybenzyl (Cbz)-protected cytosin-1-ylacetic acid formed successfully the nucleo-aminooxy ester **120**. However, the analogous reaction with thymin-1-ylacetic acid was found to be problematic because of the difficult purification. The phthaloyl group in **119** was then replaced by a Boc group to give **121** via Cbz-protection of the amino group. The Cbz group in **121** should be removed using Pd-catalyzed hydrogenolysis in less than 10 min to avoid reductive cleavage of the N-O bond. The thymidine aminooxy ester **122** was obtained in moderate yield because of the over acylation on the NH-O nitrogen. Improved yields were obtained from the *bis*-Boc-protected **123**. Subsequently, full deprotection led to the target nucleo-aminooxy acids **126** and **127**. Triazole-linked nucleo aminooxy esters **129** and **130** have been prepared from the azido derivative **128**, where the use of ascorbic acid instead of sodium ascorbate is essential to avoid the elimination of the phthalimidooxy moiety during the triazole formation.

**Scheme 18:**
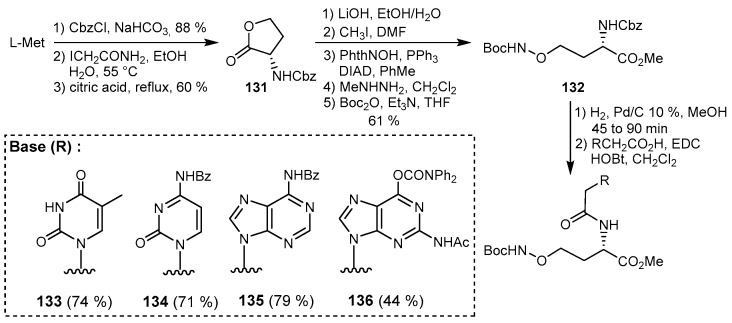
Synthesis of nucleo γ-aminooxy acid derivatives.

However, synthesis of the corresponding nucleopeptides was hampered by the rapid elimination reaction of the phthalimidooxyl group to give an acylamidoacrylate derivative during the deprotection of methyl esters in **124**, **125**, **129** and **130**. To avoid this side reaction, we have then decided to prepare nucleo-aminooxy acids based on α-amino-γ-aminooxy ester **132**, which can be obtained from *N*-Cbz-l-Met through *S*-alkylation with iodoacetamide followed by lactonisation in hot citric acid (Scheme 18) [43]. To remove the Cbz protecting group without the cleavage of the N-O bond, 40% Pd/C (*w*/*w*) was necessary to quickly deprotect the Cbz group. Subsequent coupling with functionalized nucleobases afforded the corresponding nucleo γ-aminooxy esters **133**–**136**. 

**Scheme 19:**
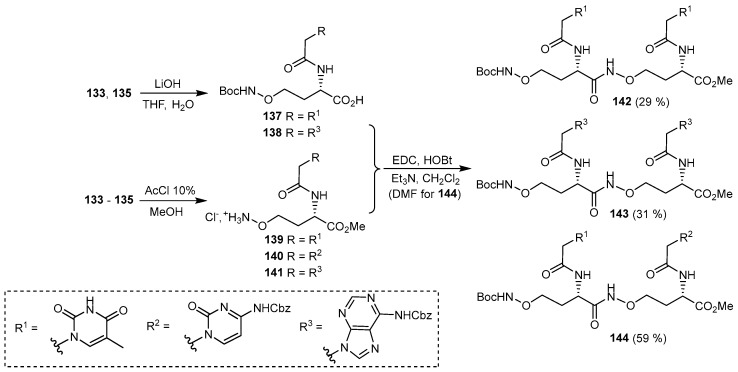
Synthesis of *N*-oxyamide-linked nucleopeptides.

As expected, the obtained nucleo γ-aminooxy esters can be saponified to the corresponding carboxylic esters without elimination of the phthalimidooxyl group (Scheme 19). Coupling reaction with the oxyamine derivatives **139**–**141** furnished the *N*-oxyamide-linked nucleopeptides **142**–**144**. Improved coupling yield could be obtained by using DMF as solvent to solubilize the starting materials.

## 7. Conclusions

From readily available sugars like glucose and galactose, as well as the amino acids serine and methionine, we have developed several synthetic approaches to glyco-, glycosyl, nucleoside and nucleo-aminooxy acid derivatives. Thanks to the high reactivity of the oxyamine function, these compounds have been successfully employed as multifunctional scaffolds for the generation of *N*-oxyamide-linked oligosaccharides, glycopeptides, glycolipids, oligonucleosides and nucleopeptides as novel glycoconjugates or oligonucleotide mimics. Furthermore, the synthesized *N*-oxyamide-linked glycolipids were found to be able to form a new type of glyco-AuNP capable of sugar receptor-targeting cell imaging and drug delivery. Synthesis and derivatization of different carbohydrate aminooxy acids works well. For the synthesis of deprotected derivatives, the benzyl protecting group needs to be avoided due to the instability of N-O bonds under radical conditions. Synthesis of nucleoside aminooxy acids from the nucleosides remains challenging because of the difficulty to introduce an aminooxyl function on the sugar moiety and the decomposition problems observed during the deprotection steps. To the best of our knowledge, the *N*-glycosylation of the furanosidic aminooxy esters remains the only current method to access nucleoside aminooxy esters bearing five different nucleobases. We are convinced that these results should be useful to further development of *O*-amino sugars and nucleosides with interesting biological applications.
